# Translating antibiotic prescribing into antibiotic resistance in the environment: A hazard characterisation case study

**DOI:** 10.1371/journal.pone.0221568

**Published:** 2019-09-04

**Authors:** Andrew C. Singer, Qiuying Xu, Virginie D. J. Keller

**Affiliations:** 1 NERC Centre for Ecology & Hydrology, Benson Lane, Wallingford, United Kingdom; 2 Environmental Diagnosis and Management, Royal Holloway University of London, Egham, United Kingdom; University of Maryland School of Medicine, UNITED STATES

## Abstract

The environment receives antibiotics through a combination of direct application (e.g., aquaculture and fruit production), as well as indirect release through pharmaceutical manufacturing, sewage and animal manure. Antibiotic concentrations in many sewage-impacted rivers are thought to be sufficient to select for antibiotic resistance genes. Yet, because antibiotics are nearly always found associated with antibiotic-resistant faecal bacteria in wastewater, it is difficult to distinguish the selective role of effluent antibiotics within a ‘sea’ of gut-derived resistance genes. Here we examine the potential for macrolide and fluoroquinolone prescribing in England to select for resistance in the River Thames catchment, England. We show that 64% and 74% of the length of the modelled catchment is chronically exposed to putative resistance-selecting concentrations (PNEC) of macrolides and fluoroquinolones, respectively. Under current macrolide usage, 115 km of the modelled River Thames catchment (8% of total length) exceeds the PNEC by 5-fold. Similarly, under current fluoroquinolone usage, 223 km of the modelled River Thames catchment (16% of total length) exceeds the PNEC by 5-fold. Our results reveal that if reduced prescribing was the sole mitigating measure, that macrolide and fluoroquinolone prescribing would need to decline by 77% and 85%, respectively, to limit resistance selection in the catchment. Significant reductions in antibiotic prescribing are feasible, but innovation in sewage-treatment will be necessary for achieving substantially-reduced antibiotic loads and inactivation of DNA-pollution from resistant bacteria. Greater confidence is needed in current risk-based targets for antibiotics, particularly in mixtures, to better inform environmental risk assessments and mitigation.

## Introduction

The environment receives antibiotics through a combination of direct application (e.g., aquaculture and fruit production), as well as indirect release through pharmaceutical manufacturing, sewage and animal manure [1,2]. As part of a One Health approach, the global agenda aims to reduce antibiotic use and misuse in human, animal and agriculture with downstream benefits to the environment [3–6]. To this end, in 2013 Professor Dame Sally Davies, the Chief Medical Officer (CMO) for England and Chief Medical Advisor to the UK government, published her Annual Report [7] in which she argued for a reduction in inappropriate prescribing of antimicrobials in the UK. In response, the National Health Service of England (NHS) proposed targeted reductions in antibiotic prescriptions as part of the Quality Premium Programme (QPP) [8].

The QPP in 2015/16 aimed to reduce antibiotic over-use and inappropriate prescribing through a reduction in:

1. the number of antibiotics prescribed in primary care by ≥1% from each Clinical Commissioning Group (CCG’s) 2013/14 value;
2. the proportion of broad-spectrum antibiotics prescribed in primary care. Specifically, the QPP aims to reduce prescriptions of co-amoxiclav, cephalosporins and fluoroquinolones by 10% (from each CCG’s 2013/14 value) as a percentage of the total number of antibiotics prescribed in primary care, or to be below the 2013/14 median proportion for English CCGs (11.3%), whichever represents the smallest reduction.

The NHS of England successfully reduced their antibiotic prescriptions in 2015/16 by 7.3% (37.03 million items to 34.34 million) as compared to 2014/15. The NHS also saw a reduction of 16% in broad-spectrum antibiotics (3.94 million items to 3.3 million). The goal for 2016/17 was, in part, to reduce total antibiotic prescribing in primary care by 4% (based on 2013/14) and broad-spectrum antibiotics by 20% (based on 2014/15). The majority of the reductions seen in 2016/17 were for amoxicillin, co-amoxiclav, and some cephalosporins, not macrolides or fluoroquinolones. The only exceptions being erythromycin, which saw a reduction in consumption in the primary care setting, of 0.078 DDDs and azithromycin which saw an increase of 0.023 DDDs per 1000 inhabitants per day in England [9].

Any reduction in antibiotic prescribing would result in a proportional reduction in antibiotics released into wastewater. This is because a significant fraction of antibiotics are conserved as they pass through the body before being excreted as a mixture of the parent compound and metabolites in the urine and faeces [10,11]. The gut bacteria from hundreds of thousands of antibiotic-consuming NHS patients would have been enriched in antibiotic resistance, a phenomenon that is unavoidable upon consumption of antibiotics [12], and released along with the antibiotics into the receiving river. Antibiotic concentrations in many sewage-impacted rivers are thought to be sufficient to select for antibiotic resistance genes [13–18]. However, because antibiotics are nearly always found associated with antibiotic-resistant faecal bacteria in wastewater, it is difficult to distinguish the selective role of effluent antibiotics within a ‘sea’ of gut-derived resistance genes [19,20]. The effects of this phenomena are most evident downstream a sewage treatment plant (STP) discharge point as compared to upstream, for which examples can be found globally [20–25].

This study focused on two questions that explore the link between antibiotic use and environmental impact:

1. To what extent might current macrolide and fluoroquinolone prescribing contribute to antibiotic resistance selection in sewage-impacted rivers in southern England?
2. How much of a reduction in macrolide and fluoroquinolone prescribing might be required to alleviate the hazard of antibiotic resistance selection in rivers, if it was the sole means of mitigating environmental load?

The study focused on macrolides and fluoroquinolones because: 1) they are two of the more persistent classes of antibiotics *in vivo* and the environment, and as such, are found in nearly all antibiotic surveillance studies [26–31]; 2) they have significant clinical relevance [32]; and, 3) macrolides are on the EU Watch List of Decision 2015/495/EU [33,34].

The River Thames catchment (i.e., Thames Basin) in southern England was selected for this study as it is the most highly populous catchment in the United Kingdom (nearly 4 million people). It also might be seen as a realistic worst-case scenario, as this part of England is in the 25^th^ quartile of predicted values of annual dilution factors, i.e., between 2.24 and 6.26 [35]. Many river stretches within the catchment offer little opportunity for dilution, maximising the impact of antibiotics found in the discharged sewage. Furthermore, the catchment has previously been the subject of several studies investigating predicted environmental concentrations of pharmaceuticals [30,36–38].

## Materials and methods

### Prescription data

**Fig 1:**
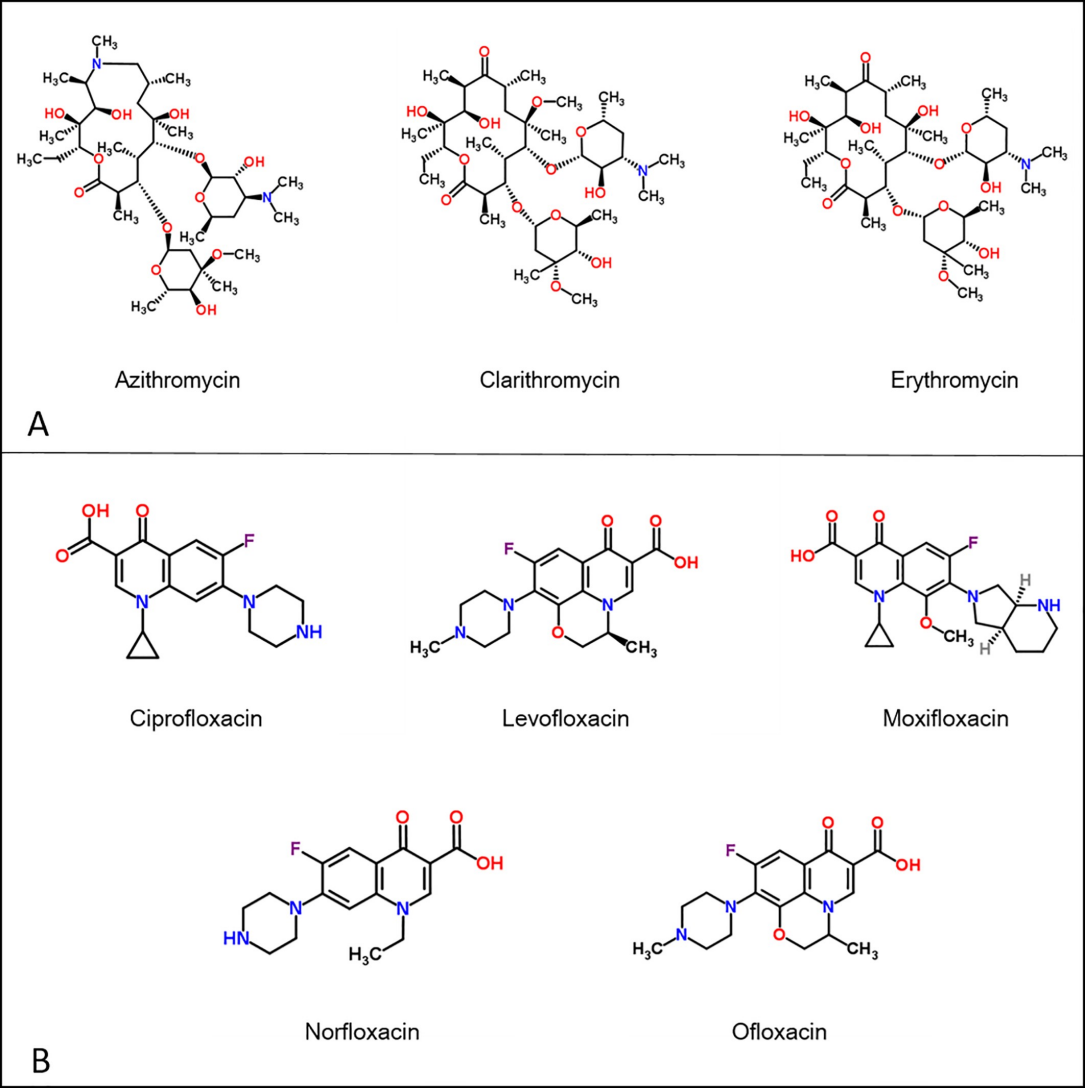
Macrolides (A) and fluoroquinolones (B) included in the study.

**Fig 2:**
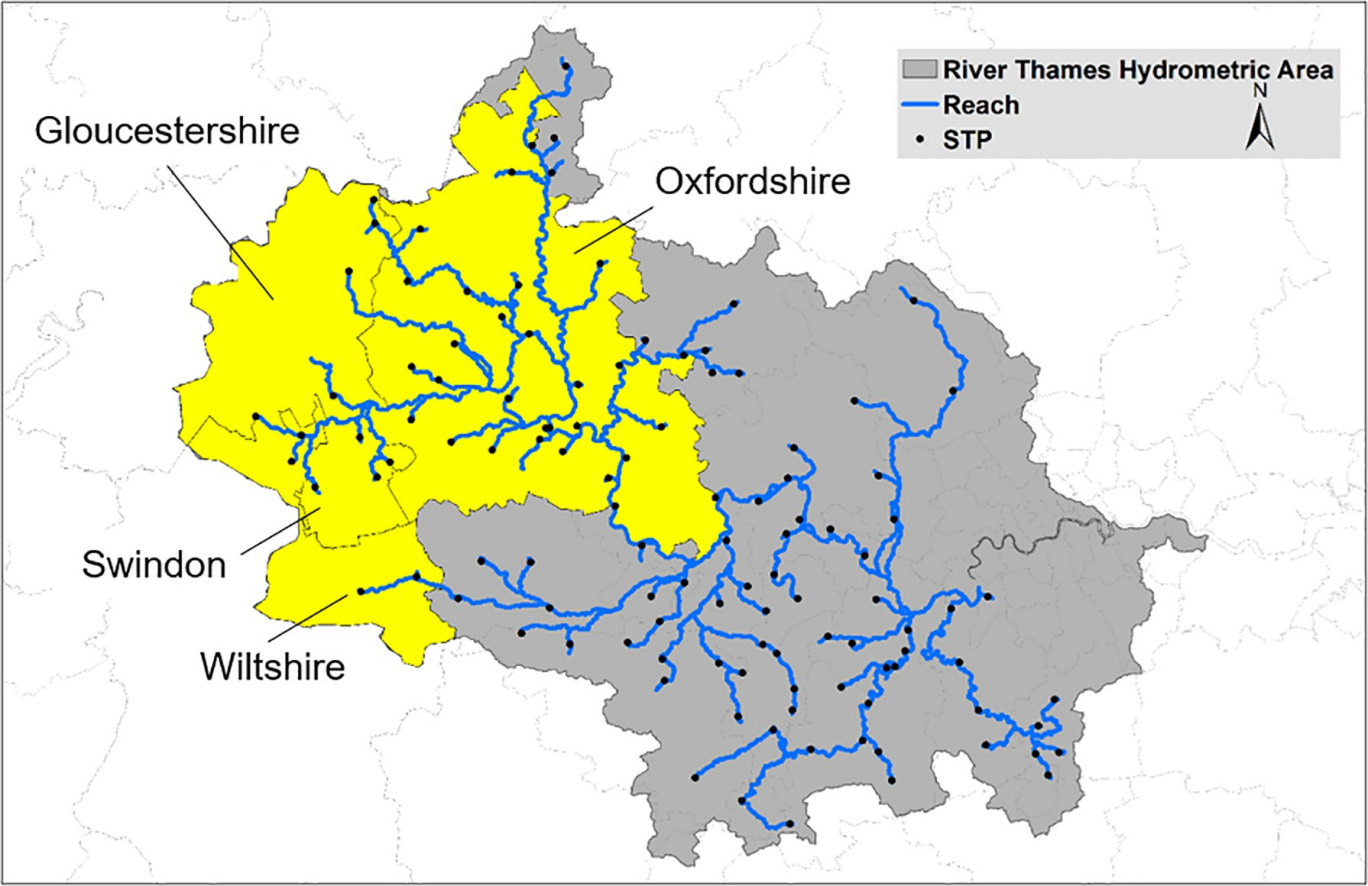
Hydrometric area of Thames Region with the four CCGs in the upper Thames. Catchment denoted in yellow.

Macrolide prescribing data for each month of 2015/16 were acquired from the NHS Business Service Authority (NHSBSA), i.e., azithromycin, clarithromycin and erythromycin (Fig 1A). Fluoroquinolone prescribing data for each month of 2015/16 were also acquired from the NHSBSA, i.e., ciprofloxacin, levofloxacin, moxifloxacin, norfloxacin and ofloxacin (Fig 1B). Prescription data were acquired from four Clinical Commissioning Groups (CCGs): Oxfordshire, Gloucestershire, Swindon and Wiltshire, all of which are situated in the upper River Thames catchment (Fig 2). It was not possible to reliably assign CCG prescriptions to STPs in the lower Thames catchment owing to the small geographic size of the CCGs and high population density. As such, all other CCGs within the catchment were assigned the prescription rate of NHS Oxfordshire CCG.

NHS prescription data for each CCG per month in 2014/15 were converted to units of kg (Table 1), and then moles (Table 2) of macrolide and fluoroquinolone and subsequently normalised to the population within each CCG (Table 3). In the case of erythromycin, the three forms of erythromycin (erythromycin, erythromycin ethyl succinate and erythromycin stearate) were converted individually and summed.

**Table 1:** Mass of macrolides and fluoroquinolones prescribed in the study CCGs in 2015/16.

		Oxfordshire (kg)	Gloucestershire (kg)	Swindon (kg)	Wiltshire (kg)
Macrolides	Azithromycin	21.8	18.9	4.5	12.6
	Clarithromycin	179.5	322.7	66.7	198.0
	Erythromycin	97.3	80.7	50.6	82.3
	Erythromycin ethyl succinate	120.8	79.0	32.3	34.4
	Erythromycin stearate	12.4	5.4	4.3	5.0
Total of macrolides (kg)		431.8	506.7	158.4	332.3
Fluoroquinolones	Ciprofloxacin	93.8	94.6	29.5	72.2
	Levofloxacin	0.3	4.6	0	0.3
	Moxifloxacin	0.5	0.1	0.1	0.2
	Norfloxacin	0	0	0.1	0
	Ofloxacin	2.2	4.1	0.7	1.0
Total of fluoroquinolones (kg)		96.8	103.4	30.4	73.7

**Table 2:** Molecular weights of macrolides and fluoroquinolones.

Macrolides	Molecular weight (g/mol)	Quinolones	Molecular weight (g/mol)
Azithromycin	748.98	Ciprofloxacin	331.34
Clarithromycin	747.95	Levofloxacin	361.36
Erythromycin	733.90	Moxifloxacin	401.43
Erythromycin ethyl succinate	862.10	Norfloxacin	319.33
Erythromycin stearate	1018.40	Ofloxacin	361.36

**Table 3:** Moles of macrolides (M) and fluoroquinolones (F) prescribed capita^-1^ day^-1^ month^-1^ within each CCG.

CCGs	Oxfordshire^1^		Swindon^2^		Gloucestershire^3^		Wiltshire^4^	
Population	666100		231277		624000		500000	
Antibiotics	M	F	M	F	M	F	M	F
	×10^−7^ mol							
January	**30.4**	**13.3**	24.8	**12.5**	27.9	12.7	23.4	11.5
February	28.7	12.8	28.4	11.2	36.7	**16.4**	**30.2**	**13.9**
March	28.1	11.7	**30.5**	11.8	**36.8**	15.5	28.0	11.8
April	23.6	11.9	26.0	11.1	30.4	12.5	25.8	11.8
May	21.3	11.1	22.8	9.50	26.9	13.6	23.1	10.9
June	22.5	12.0	25.6	12.9	28.8	13.1	24.2	11.8
July	20.5	11.5	24.8	11.3	26.7	13.6	22.5	12.3
August	10.2	11.6	18.2	9.40	22.0	12.0	19.9	12.4
September	20.9	11.2	22.3	9.30	25.2	13.7	22.3	12.4
October	22.0	12.3	22.1	10.9	27.8	13.6	22.3	12.8
November	22.2	12.1	22.1	9.80	29.1	12.5	21.6	11.4
December	23.4	12.6	25.4	10.0	31.8	14.4	25.6	12.9

Population statistics are provided by the respective Annual Report for the CCG. Bolded values in each column represent the month with the maximum prescription rate in 2015. These were selected for use in the model to ensure a realistic worst-case scenario.

^1^ NHS Oxfordshire CCG Annual Report

^2^ NHS Swindon CCG Annual Report

^3^ NHS Gloucestershire CCG Annual Report

^4^ NHS Wiltshire CCG Annual Report

### Antibiotic excretion rate

The excretion of antibiotics in the faeces and urine depends on its metabolism *in vivo*, which is a function of health, age, gender, ethnicity, as well as dosage and mode of administration, e.g., capsule, injection. Given the large uncertainty associated with estimating excretion rates across diverse populations, an average rate acquired from the literature was used: macrolide excretion rate of 32.2% and 64.2% for fluoroquinolones (Table 4). These rates are consistent with excretion rates used in Besse *et al*. 2008 [39], which were subsequently used to inform the “Development of the first Watch List under the Environmental Quality Standards Directive”[40]. Excretion rates are inclusive of well characterised conjugated metabolites of antibiotics that can be ‘reactivated’ in the environment. For example, the urinary and biliary elimination of ciprofloxacin as metabolites respectively represents 12.5 and 2.3% of the dose (total: 14.8%), only a fraction of which is likely to be reactivated in the environment [41].

**Table 4:** Human excretion of fluoroquinolones and macrolides as a percentage of the parent compound.

Class of Antibiotic	Antibiotics	Excretion (% of parent compound)
Fluoroquinolones	Ciprofloxacin	53.8 [42], 40 [43], 50 [11], 45–60 [44]
	Levofloxacin	71 (60–80) [42], 70 [43]
	Moxifloxacin	60 [45]
	Norfloxacin	61.5 [42], 30 [43]
Macrolides	Ofloxacin	75.8 (60–80) [42], 100 [11]
	Azithromycin	50 [11], 50 [44], 8 [43]
	Clarithromycin	33.7 (14.4–60) [42], 20 [43], 0.18 [11]
	Erythromycin	35 (3.5–98) [42], 8 [43], 12–15 [44]

### Antibiotic loss in STPs

The antibiotic loss in STPs was modelled using STPWIN model within the Estimation Program Interface (EPI) Suite^TM^ 4.0, using the Biowin/EPA draft method for determining half-life data as previously described [37]. Estimates of loss in STPs was also acquired from the literature [46–51], with some relevant data acquired from STPs within the River Thames catchment found in Table 5. These data support the use of 50% loss during STP passage, which is a compromise between measured loss and the high variability reported within the literature.

**Table 5:** Measured antibiotics concentration in STPs and rivers within the Thames Catchment (ng/L) [52].

	Macrolide			Fluoroquinolone		
	CLAR	ERY	AZO	CIP	NOR	OFL
Eff–Oxford*	98 (338–1504)		156 (69–264)			
Eff–Didcot*	104 (160–305)		110 (74–216)			
Eff–Cholsey*	181 (128–321)		91 (48–135)			
Eff–Benson*	152 (87–254)		76 (35–133)			
Eff–Benson**	50	244	34	14	25	195
Eff–Oxford**	91	236	30	52	21	23
Thames var.***	292, 30	448, 58	51, 21	46, 20	45, 9	17, 11

Eff sewage effluent, CLAR clarithromycin, ERY erythromycin, AZO azithromycin, CIP ciprofloxacin, NOR norfloxacin, OFL ofloxacin

* Mean (highest–lowest) measured

**24-h mean concentration [30]

***Max and mean concentration for 21 locations at 7 time points [30]

### Modelled environmental concentrations of antibiotics

Low Flows 2000 Water Quality eXtension (LF2000-WQX) model [38,53] is an extension to the LF2000 [54]. LF2000 is a decision support tool designed to estimate river flow at gauged and ungauged sites and to assist regional water resources and catchment management. The LF2000-WQX software is a geographical information-based system that combines hydrological models with a range of water-quality models, including a catchment-scale water-quality model. This model generates spatially explicit statistical distributions of down-the-drain chemicals for both conservative and degradable compounds. It uses a Monte Carlo model approach to combine statistical estimates of chemical loads at specific emission points (e.g., STP) with estimated river flow distributions for the whole river network of interconnected model reaches (a reach is the river stretch between model features, e.g., major tributaries, STPs). The hydrometric area of the River Thames catchment and the STPs included in LF2000-WQX (black points) can be seen in Fig 2. Thus, working from the upstream reaches at the head of the river network to the outlet of the river basin, the model accounts for the accumulation of point antibiotic loads and the water in which these loads are diluted. Degradable chemicals might be removed from STPs and the river water. The latter is represented by a non-specific dissipation process assuming first-order kinetics. Antibiotics were assumed to persist in the river for at least one day, thereby providing a realistic worst-case scenario.

In summary, LF2000-WQX was parameterised with the highest monthly fluoroquinolone and macrolide prescription rate for each of the four CCGs (see bolded rates in Table 4). Macrolide and fluoroquinolone loss was accounted for upon excretion (32.2% and 64.2%, respectively) and before discharge from STPs into the receiving river (50%). As such, the mass of macrolide and fluoroquinolone consumed was reduced by 83.9% and 67.9%, respectively, upon discharge into the adjacent river. The antibiotic load was subsequently diluted within the river, parameterised with the mean annual river flows within LF2000-WQX.

### Risk-based targets for antibiotics

Risk-based management targets for antibiotics in freshwater would ideally be set at concentrations that are below the lowest concentration that allows antibiotics to select for antibiotic resistance genes, referred to as a Predicted No Effect Concentration for selection (PNEC) [55]. Lowest effect concentrations have been determined experimentally in a limited number of lab-based studies, operationally termed minimum selection concentrations (MSC). MSCs typically range between 0.1 to 10 μg of antibiotic/L [14,16,17,55–64].

Modelled PNECs were acquired from Bengtsson-Palme and Larsson (2016) owing to the absence of empirically-determined MSCs for most of the study antibiotics. The PNECs derived in Bengtsson-Palme and Larsson (2016) were selected to inform antibiotic discharge limits from pharmaceutical manufacturing by the AMR Industry Alliance [65]. The PNECs represent the threshold concentration of an antibiotic, above which, there is a heightened hazard of antibiotic resistance selection. PNECs were derived from the European Committee on Antimicrobial Susceptibility Testing (EUCAST) database of minimum inhibitory concentrations to form species sensitivity distributions [37]. The authors selected the concentration of each antibiotic representing the 1% potentially affected fraction (PAF). A safety factor of 10 was added to this 1% PAF to account for the observation that experimentally-derived resistance selection thresholds tend to be approximately an order of magnitude lower than the MIC, while also offering an added level of protection to the estimate. The 111 antibiotic thresholds reported in Bengtsson-Palme and Larsson 2016 ranged from 0.008 μg/L to 64 μg/L.

PNECs for fluoroquinolones ranged from 0.064 to 0.5 μg/L and for macrolides 0.25 to 1 μg/L (Table 6). When predicted environmental concentrations (PECs) of macrolides and fluoroquinolones exceeded the PNEC for the most ‘potent’ antibiotic within each class, e.g., ciprofloxacin for fluoroquinolones and azithromycin/clarithromycin for macrolides, the stretch of river was denoted as being ‘at risk’ for resistance selection. When the PEC > PNEC for the least ‘potent’ antibiotic within each class, e.g., norfloxacin/ofloxacin for fluoroquinolones and erythromycin for macrolides, the stretch of river was denoted as being at a ‘critical risk’ of resistance selection.

**Table 6:** Modelled PNECs for macrolides and fluoroquinolones [55].

Antibiotic class	Antibiotic	PNEC (μg/L)	PNEC (10^−10^ mol/L)
Fluoroquinolones	Ciprofloxacin	0.064	1.9
	Levofloxacin	0.25	6.9
	Moxifloxacin	0.125	3.1
	Norfloxacin	0.5	15.7
	Ofloxacin	0.5	13.8
Macrolides	Azithromycin	0.25	3.3
	Clarithromycin	0.25	3.3
	Erythromycin	1	9.8

## Results

This study aimed to understand the degree of resistance selection that might be occurring in the River Thames catchment under current macrolide and fluoroquinolone prescribing practice by the NHS as well as after foreseeable and aspirational reductions in prescribing. The objective was to estimate the level of reduction in NHS antibiotic prescribing that might be necessary to reduce the hazard of resistance selection in sewage-impacted rivers.

**Fig 3:**
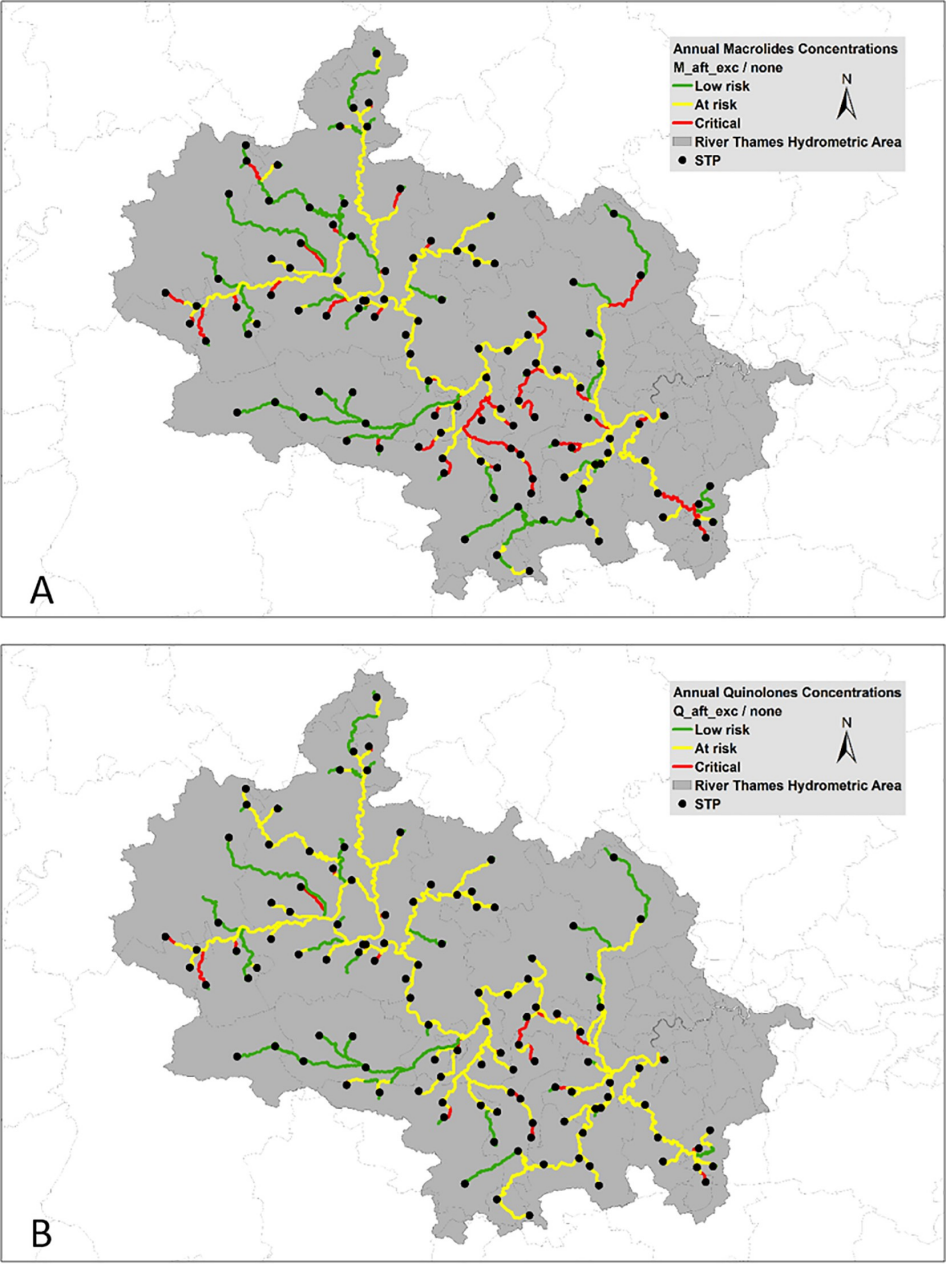
Resistance selection risk characterisation for (A) macrolides and (B) fluoroquinolones using 2015/16 prescription statistics.

The maxima PECs for macrolides and fluoroquinolones prescribed in 2015/16 were 4.3 μg/L and 2.0 μg/L, respectively. Hazard characterisation revealed 279 out of 457 reaches in the catchment are ‘at risk’ or ‘critical’ for macrolide resistance selection (Fig 3A)—equating to 1155 of 1398 km of the modelled River Thames catchment (64.0% of the total catchment length modelled). Similarly, 311 reaches out of 457 were ‘at risk’ or ‘critical’ for fluoroquinolone resistance selection (Fig 3B)—equating to 1026 of 1398 km (73.7% of the modelled river length in the catchment).

Table 7 contains the sum (km) and fractional river length that exceeds the PNEC for the most and least ‘potent’ macrolide and fluoroquinolone; operationally defined as ‘critical’ and ‘at risk’ of resistance selection, respectively. The length and % of river lengths that exceed multiples (2-5x) of the PNEC are also presented. Such analysis reveals that under current macrolide usage, 115 km of the modelled River Thames catchment exceeds the PNEC for ‘at risk’ by 5-fold (8% of modelled catchment length; Table 7). Similarly, under current fluoroquinolone usage, 222.9 km of the modelled River Thames catchment exceeds the PNEC for ‘at risk’ by 5-fold (16% of modelled catchment length). Nearly one-fifth of the catchment length exceeds the macrolide PNEC for ‘critical’ under current usage, whereas fluoroquinolones exceed the PNEC for ‘critical’ in only 5% of the catchment length. Macrolide levels in the river exceed double the ‘critical’ PNEC in 5% of the catchment length, whereas fluoroquinolones, at such levels, are exceeded in <1% of the catchment length (Table 7).

**Table 7:** Sum and fractional river length exceeding the PNEC and multiples of the PNEC for macrolides and fluoroquinolones.

	Selection Hazard	PNEC (10^−10^ mM)	Multiple of PNEC				
Macrolide			1x	2x	3x	4x	5x
			River length (km)				
	Sum > at risk	3.3	895.0	469.0	266.2	174.5	115.5
	Sum > critical	9.8	270.3	75.5	16.3	3.7	0.4
			River length (%)				
	% > at risk	3.3	64.0%	33.6%	19.1%	12.5%	8.26%
	% > critical	9.8	19.3%	5.41%	1.17%	0.26%	0.03%
**Fluoroquinolone**			River length (km)				
	Sum > at risk	1.9	1030.2	724.1	473.4	351.1	222.9
	Sum > critical	15.7	81.6	11.1	0.1	0.0	0.0
			River length (%)				
	% > at risk	1.9	73.7%	51.8%	33.9%	25.1%	16.0%
	% > critical	15.7	5.84%	0.79%	0.01%	0.00%	0.00%

Consistent with the 2015/16 QPP goals, a 4% reduction in macrolide prescribing saw a 3.8% reduction in the length of the modelled Thames catchment ‘at risk’ for macrolide resistance selection (273 reaches and 830 km, equal to 59.4% of the catchment), and a 0.1% reduction in the length of the modelled Thames catchment ‘at risk’ for fluoroquinolone resistance selection (310 reaches and 1025.1 km, equal to 73.3% of the catchment).

A more ambitious 20% reduction of the 2015/16 prescription rate of macrolides resulted in an 8.9% reduction in the length of the modelled Thames catchment ‘at risk’ for macrolide resistance selection (254 reaches and 759 km, equal to 54.3% of the catchment). A 20% reduction in fluoroquinolones resulted in a 5.4% reduction in the length of the modelled Thames catchment ‘at risk’ for fluoroquinolone resistance selection (296 reaches and 949 km, equal to 68.0% of the catchment).

A sensitivity analysis was conducted to identify the NHS prescribing rate that protects ≥90% of the length of the modelled Thames catchment from macrolide and fluoroquinolone resistance selection. A target of 90% was a pragmatic decision, as many rivers in England are highly impacted by, or solely composed of, sewage effluent—particularly in headwaters. Hence, a target of 100% would be unrealistic and necessitate even more dramatic reductions in antibiotic use or zero effluent discharge.

**Fig 4:**
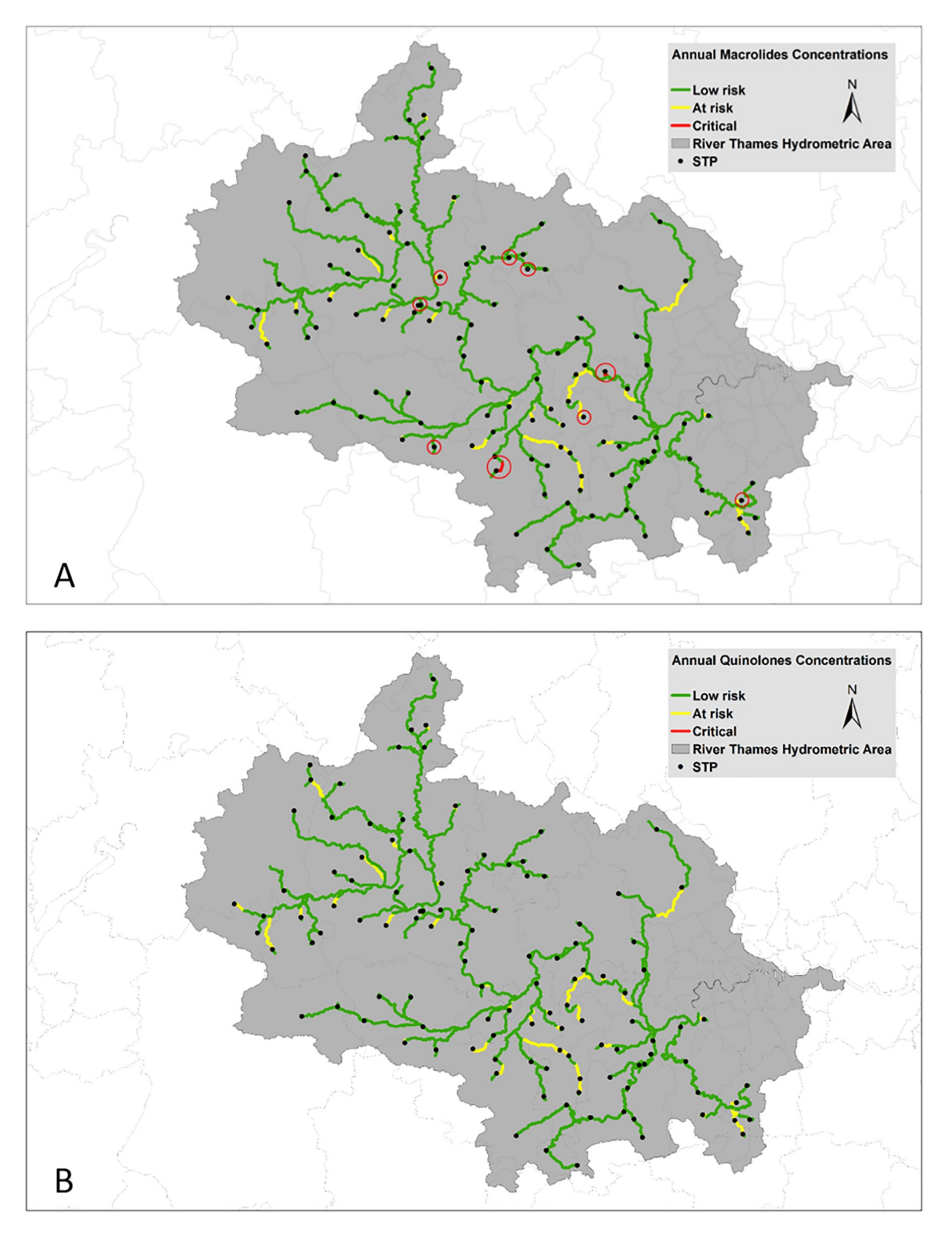
Hazard characterisation for (A) macrolide and (B) fluoroquinolone resistance selection after a reduction of 77% and 85% in prescriptions, respectively, on 2015/16 rates. Red circles in (A) indicate nine locations where the concentrations remained ‘at critical’ levels.

The sensitivity analysis revealed that macrolide prescriptions must decline by 77% to protect 90.4% of the length of the Thames catchment from macrolide resistance selection (Table 8; Fig 4A). Even at this much-reduced prescription rate, there were still 13 reaches with a total length of 8.5 km (0.6%) remaining ‘critical’ for macrolide resistance selection (Fig 4A). Fluoroquinolone prescriptions would need to be reduced by 85% to alleviate the selection risk in 90% of the catchment (Table 8; Fig 4B).

**Table 8:** Sensitivity analysis for the level of reduction in prescriptions needed to protect 90+% of the length of the modelled River Thames catchment from resistance gene selection.

	% Reduction in Prescriptions	Number of reaches ‘at risk’ or ‘critical’	Length ‘at risk’ or ‘critical’ (km)	% Length ‘at risk’ or ‘critical’
Macrolides	80	69	110	7.9
	**77**	**82**	**134**	**9.6**
	76	86	157	11.2
	75	93	169	12.1
Fluoroquinolones	**85**	**82**	**135**	**9.6**
	84	88	157	11.2
	83	96	175	12.5
	80	118	215	15.4

## Discussion

The goal of this study was to evaluate how antibiotic prescribing, alone, contributes to antibiotic resistance selection in the aquatic environment. Such a perspective is fundamentally naïve as the catchment contains considerable land for grazing and is at risk of receiving animal waste containing antibiotics. Pollutants found in sewage, agriculture, and street runoff can co-select for antibiotic resistance, making this model a conservative estimate of the hazard of resistance selection [66–68].

A non-trivial proportion of the resistance genes found downstream of STPs originate from human gut bacteria, many of which have acquired resistance *in vivo*, perhaps during antibiotic treatment of human infections [69–71]. Antibiotic-resistance genes disseminated from anthropogenic sources into the environment are pollutants [72,73] maintained and spread through a combination of mechanisms: 1) survival of enteric bacteria in the environment and wildlife [74–76]; 2) horizontal gene transfer of resistance genes from enterics into indigenous environmental bacteria [77]; 3) selection and co-selection taking place *in situ* [17,78,79] and 4) transformation of extracellular DNA (eDNA) containing antibiotic resistance genes into environmental bacteria [80]. In environments naïve to antibiotics, antibiotic exposure selects for overgrowth of resistant bacteria, while simultaneously selecting for the assembly and evolution of complex genetic vectors encoding, expressing, linking, and spreading that and other resistance genes throughout the microbial population [81]. Once evolved, a competitive construct can spread globally, such as the plasmid-borne mcr-1 [82] and bla_CTX-M-14_ [83]. Dissemination of mcr-1 and bla_CTX-M-14_, in particular, has been attributed to conjugative plasmids rather than to clonal expansion of a bacterial host strain owing to the high level of clonal diversity [83–85]. Conversely, in environments not naïve to antibiotics, where antibiotic exposure is chronic, might not experience an ‘overgrowth of resistant bacteria’ as the microbial community is already well-adapted to such chemical challenges. Arguably, such areas where there is chronic antibiotic exposure and the luxury of time (i.e., sewage-impacted rivers), there is an elevated hazard of novel evolutionary adaptations, e.g., novel gene assembly, epistasis and compensatory mutations, that can address the reduced fitness costs associated with harbouring antibiotic resistance genes [86,87].

Evidence of antibiotic-resistant microorganisms recovered from the River Thames catchment has been reported in the literature. Djanji *et al*. 2011, isolated extended-spectrum β-lactamase-producing *Escherichia coli* (bla_CTX-M-14_) belonging to the clinically important O25b:H4-ST131 lineage from the River Thames in west London [88]. Amos *et al*. 2015, analysed sediment samples from thirteen sites across the River Thames basin for class 1 integron prevalence and cefotaxime- (third-generation cephalosporin)-resistant *E*. *coli* [21]. The authors reported antibiotic resistance was strongly correlated to the proximity and size of upstream STPs. Lehmann *et al*. 2016, showed a rapid increase in class 1 integrons (*intI1*) in natural river biofilms, within the Thames catchment, exposed to trace levels of additional STP effluent [89]. The statistically significant increase in *intI1* reported within sewage-effluent-exposed periphyton occurred without statistically significant changes in the microbial community. This result was suggestive of increased horizontal gene transfer—a phenomenon that is important for the dissemination of antibiotic resistance genes. Using a novel Raman-Deuterium Isotope Probing technique, Song *et al*. 2017 reported 35±5%, 28±3%, 25±1% of the total bacterial population recovered from the River Thames with resistance to carbenicillin-, kanamycin- and both antibiotics, respectively [90]. The use of a non-selective growth medium (D_2_O) and minimally-destructive instrument for resistance detection (Raman) thereby minimises the culture bias usually introduced into such determinations, potentially offering a more realistic measure of the prevalence of antibiotic resistance within the microbiota of the River Thames.

### Strengths and limitations

To our knowledge, this study is the first attempt to model the impact of current and aspirational antibiotic prescribing in England on antibiotic resistance selection in freshwater rivers. The study’s approach reflects the state of our knowledge at this time, and as such, is limited in many ways.

#### Heterogeneity of population

The results do not reflect the granular differences in prescribing that are seen between small CCGs as there would be no way to attribute an antibiotic user to a particular STP confidently. It is expected that if such an effort were possible, it would highlight elevated antibiotics in STPs that have: older demographics (e.g., care homes [91]), hospitals [92,93], and areas of inhabitants with elevated international travel [94,95]. Should these STPs be located at or near headwaters, it would likely result in elevated hazards for antibiotic resistance selection resulting from lower effluent dilution. Conversely, when these high-antibiotic users are located in stretches where there is sufficient dilution, the selection hazard would be alleviated, somewhat.

#### Varied antibiotic stewardship within antibiotic class

In this model, we applied any reductions in antibiotic prescribing, i.e., 4 or 20%, uniformly across all antibiotics within the class. However, the use of antibiotics within a class are unlikely to be uniformly reduced. As a result, some reductions in prescribing could delay or hasten the time to achieve ‘low risk’. For example, ciprofloxacin is the most ‘potent’ fluoroquinolone in its class, with a PNEC of 64 ng/L; ofloxacin and norfloxacin are less ‘potent’, with a PNEC of 500 ng/L. In other words, ciprofloxacin is 7.8 times more potent than ofloxacin and norfloxacin. Hence, the benefit gained from reducing 1 unit of ciprofloxacin necessitates a reduction in 7.8 units of ofloxacin or norfloxacin. As ciprofloxacin represents the majority of fluoroquinolone prescribed (96.9%; Table 2), any reduction in ciprofloxacin is likely to have the maximum impact on fluoroquinolone resistance selection.

Similarly, macrolides azithromycin and clarithromycin have PNECs of 250 ng/L, four times more potent than erythromycin (1000 ng/L). Prescription of macrolides varies considerably between the four study CCGs (Tables 2 and 4), with clarithromycin (Gloucestershire and Wiltshire CCG) or erythromycin (Oxfordshire and Swindon) being the most frequently prescribed macrolide. Hence, the CCGs that are overprescribing clarithromycin will find reduced macrolide prescriptions potentially more impactful owing to the higher potency of clarithromycin relative to erythromycin.

#### Reliability of PNEC

The PNECs employed in this study have recently emerged as modelled estimates for assessing the hazard of resistance selection [55]. To date, modelled PNECs are consistent with the growing literature base [14,16,17,55–64], and as such, have been adopted by the AMR Industry Alliance for establishing regulatory thresholds in antibiotic manufacturing waste effluent [65]. There is a risk that the modelled PNECs are too protective, and would encourage potentially costly and unnecessary mitigation. Conversely, modelled estimates of single antibiotics might also underestimate selection risk, as antibiotic (pollutant) mixtures can act synergistically, as discussed below. The academic community will need to continually challenge the suitability of these PNECs and ensure they are fit for purpose.

#### Mixture effects

Recent experimental evidence suggests antibiotics can act synergistically and antagonistically when in mixtures [96,97]. Tekin *et al*. 2018, reported that synergistic and antagonistic interactions increased in frequency with the number of drugs in the bacteria’s environment [97]. Hence, a non-trivial number of the modelled PNECs used in this study could be over- as well as under-estimating the threshold for resistance selection when applied to sewage-impacted rivers.

Brochado *et al*. 2018, demonstrated that many synergies were found within the same class of antibiotic, in particular, where the same cellular process was targeted. The authors postulated that by targeting the same functional process at different steps, drug combinations of the same class could bypass the apparent redundancy of having multiple antibiotics within the same class and exhibit a synergistic effect [96]. The authors also demonstrated that many of the drug antagonisms involve antibiotic resistance mechanisms that modulate intracellular drug concentrations and not direct interactions of the primary drug targets. Hence, some antibiotic combinations were less inhibitory than expected (i.e., non-additive) because the cell’s response to one drug helped to buffer the effects from the second drug. For example, a decrease in the intracellular concentration of one drug by an efflux pump can potentially also decrease the intracellular concentration of the interacting drug [96]. Notably, the authors found that the drug-drug interactions were often conserved within species (70%), but with 13–32% of the interactions strain-specific, and poor conservation across species (5% of drug-drug interactions). It is premature to apply any specific drug-drug interactions to this model, but future efforts should attempt to account for mixture effects as part of a holistic hazard assessment.

#### Mixture effects with non-antibiotics

Another limitation of this study is that it does not take into account the presence of many potentially relevant chemicals that are known to be present in sewage and sewage-impacted rivers, such as:

1. antibiotics from agriculture and animal use [98];
2. biocides, which have been shown to co-select for antibiotic resistance genes [99–101];
3. metals, which have been shown to co-select for antibiotic resistance genes [102–105];
4. other classes of pharmaceuticals that might have synergistic or antagonistic impacts on resistance selection [96,106,107] and
5. herbicides and pesticides that have been shown to co-select for resistance genes [108,109].

It is impractical to experimentally test all the chemicals found in sewage in isolation and mixtures for the threshold concentration that allows for resistance selection, as there are simply too many. However, our environment can be assayed for such insight, by measuring the diversity and quantity of antibiotic resistance genes in the river environment and their relationship to STPs, farms, hospitals, aquaculture, urban runoff, etc. Where ‘pristine’ environments still exist, they might serve as benchmarks for qualifying and quantifying ‘natural’ resistance gene prevalence and their biogeography. However, this might prove difficult as wildlife have been implicated in the dissemination of human clinical resistance genes, making ‘pristine’ locations potentially unattainable [75]. Quantifying ‘natural’ resistance gene abundance and prevalence remains critical for hazard assessment and establishing mitigation targets.

#### LF2000-WQX limitations

LF2000-WQX generates PECs from STPs characterised by dry weather flows of >5000 m^3^/d. A majority of the small STPs not included in the model are located in the upper-most reaches of the catchment where flows are typically very low. Headwaters impacted by small STPs are likely to have a disproportionate impact on seeding the catchment with pharmaceuticals as well as antibiotic-resistant bacteria, the former of which would further add to the total load of macrolides and fluoroquinolones in the catchment. The omission of these STPs is unlikely to increase the antibiotic load in the catchment substantially; however, it might impact the DNA ‘seeding’ and maintenance of antibiotic resistance.

#### Aspirational reductions in prescribing

In this study, we projected a decline in antibiotic prescribing without considering the likely change in other important factors that would occur concurrently with a gradual reduction in prescribing. An increasing and ageing UK population will consume more pharmaceuticals per capita, increasing the antibiotic load. Dryer summers, resulting from climate change, might impact the frequency PNECs are exceeded owing to lower dilution [110,111]. More intense storms predicted in a changing climate would lead to more frequent and longer combined sewage overflows (CSOs), which deposit raw sewage into the adjacent river, thereby elevating the release of resistant bacteria, genes and chemical pollutants into the environment. CSOs, septic tanks, and farm runoff represent potentially significant sources of antibiotics and resistance genes which would benefit from being included in future models. However, such data is not currently available, representing a large knowledge gap for risk assessment.

#### Validation of PECs

Previous research using LF2000-WQX to generate PECs, e.g., steroid estrogens [38,112], glucocorticoids [113], cytotoxic chemotherapy drugs [114] and antivirals [37], provides reassurance that the river network representation within LF2000-WQX is accurate (i.e., network, flows, dilution and STPs). The modelled antibiotic concentrations from LF2000-WQX, denoted by the PNEC thresholds in Table 6, are within the same range as measured environmental concentrations detailed in Table 5, lending credibility to the model outputs.

#### Antibiotic stewardship

Reduction of antibiotics in the environment can be achieved through a range of mitigating actions, the first of which can be through improved antibiotic stewardship. The results of this study indicate that current goals for antibiotic stewardship do not go far enough [5,115]. In Europe, the Netherlands has among the most restricted antibiotic stewardship, with nearly 50% fewer antibiotics prescribed in the community/primary care sector (10.06 DDD/1000 inhabitants/d) than in the UK (19.09), in 2017 (https://ecdc.europa.eu/en/antimicrobial-consumption/database/country-overview). Macrolide prescribing in the Netherlands (1.38 DDD/1000/d) was only 47% that of the UK (2.90 DDD/1000/d). The extent to which the Netherlands still overprescribes and misprescribes macrolides would be highly instructive in defining safe limits for further reductions in macrolide prescribing. Notably, the UK has a lower fluoroquinolone prescribing rate in the community (0.44 DDD/1000/d) as compared to the Netherlands (0.75). Hence, there are important lessons to be mutually shared across Europe and more widely on how to optimise antibiotic prescribing—a point not lost on the Advisory Committee on Antimicrobial Prescribing, Resistance and Healthcare Associated Infection [116] and Public Health England [117].

The UK has reported between 8.8% and 23.1% of all systemic antibiotic prescriptions in English primary care as inappropriate, with some high prescribing practices in England capable of reducing antibiotic prescriptions by as much as 52.9% [117]. In a linked study where the authors and an expert panel explored the ‘appropriateness’ of antibiotic prescription, the authors found that substantially higher proportions of patients received antibiotics than was deemed ‘appropriate’, with the respiratory ailments showing the largest contrast between actual and ideal: acute cough (41% vs 10%, respectively), bronchitis (82%:13%); sore throat (59%:13%); rhinosinusitis (88%:11%); and acute otitis media in 2- to 18-year-olds (92%:17%) [118]. Such a reduction in prescribing will be beneficial to reducing resistance selection in humans, but, as shown in this paper, might not go far enough (Table 9).

**Table 9:** Summary of the modelled impact of antibiotic prescribing on antibiotic resistance gene selection in sewage-impacted freshwater.

Scenarios	Macrolide	Fluoroquinolone
	% of modelled catchment length >PNEC	
Antibiotic prescribing from 2015/16	64	74
4% Reduction on 2015/16	59	73
20% Reduction on 2015/16	54	68
	% reduction in prescribing required to achieve the target	
≥90% of catchment <PNEC	77	85

#### Mitigation through innovation and investment in STPs

A step-change in the way we currently handle our wastewater in needed to tackle the challenge of antibiotic and DNA pollution. One option is to set emission limits on the concentration of antibiotics, in much the same way the pharmaceutical industry has voluntarily adopted for its manufacturing supply chain [65]. The setting of strict antibiotic emissions limits will have the desired effect of greatly reducing the environmental impact of antibiotics, with, arguably, several serendipitous implications for other chemical hazards. For example, any technological solution for the treatment of wastewater that is effective in removing a wide range of antibiotics will also likely reduce the load of estrogens and estrogen-mimicking chemicals which has been a growing environmental concern for several decades [119–121]. As such, it is entirely likely that the substantial cost associated with tackling estrogens and estrogen-mimicking chemicals [122] can be shared with the challenge of reducing AMR in the environment.

Moreover, there is a rapidly growing list of chemicals that are found within sewage effluent that has been shown to select or co-select for ARGs, i.e., antiepileptics [106], biocides/disinfectants [123,124], metals [125]. Hence, engineering solutions to antibiotic removal from wastewater can spread the cost across a very wide range of pollutants, thereby alleviating a substantial range of ecotoxicological hazards, in addition to resistance selection. Spreading the mitigation costs across a range of challenges might also be more politically and socially tractable, owing to the significant cost associated with improving our treatment of wastewater.

It is relevant to highlight that any engineering solutions employed for the removal of antibiotics from sewage effluent might be ineffective in reducing the DNA pollution from antibiotic-resistant bacteria. Co-development of engineering solutions to tackle the chemical and biological drivers of ARGs in the environment is required to reduce the environmental pressure caused by our wastewater. In addition, the by-products of STP, sludge, which contains antibiotic resistance, antibiotics and non-antibiotic chemical drivers of resistance are currently amended to land, representing additional risks to humans and the environment [66], but are out of the study’s scope.

## Conclusion

This study explores the reduction in macrolide and fluoroquinolone prescribing needed to alleviate the modelled hazard from antibiotic resistance selection in sewage-impacted rivers. It is unclear if the projected reductions in antibiotic prescriptions of 77 to 85% could be achieved solely through reduced prescribing by the NHS. Moreover, it remains unexplored whether, ethically, it *should* be met through changes in prescribing. Arguably, environmental targets could be more readily achieved by a holistic, integrated AMR action plan, which constrains and optimises antibiotic prescribing, while also addressing the chronic release of antimicrobials, biocides, metals and resistance genes from STP effluent.

## References

[1] Vikesland P, Garner E, Gupta S, Kang S, Maile-Moskowitz A, Zhu N. Differential Drivers of Antimicrobial Resistance across the World. Acc Chem Res. 2019;52: 916–924. 10.1021/acs.accounts.8b00643 30848890

[2] Qiao M, Ying G-G, Singer AC, Zhu Y-G. Review of antibiotic resistance in China and its environment. Environ Int. 2018;110: 160–172. 10.1016/j.envint.2017.10.016 29107352

[3] FAO. The FAO Action Plan on Antimicrobial Resistance 2016–2020: Supporting the food and agriculture sectors in implementing the Global Action Plan on Antimicrobial Resistance to minimize the impact of antimicrobial resistance. 2016. Available: http://www.fao.org/3/a-i5996e.pdf

[4] European Commission. A European One Health Action Plan against Antimicrobial Resistance (AMR). 2017. Available: https://ec.europa.eu/health/amr/sites/amr/files/amr_action_plan_2017_en.pdf

[5] Government HM. Tackling antimicrobial resistance 2019–2024: The UK’s five-year national action plan. HM Government; 2019 1 Available: https://assets.publishing.service.gov.uk/government/uploads/system/uploads/attachment_data/file/784894/UK_AMR_5_year_national_action_plan.pdf 10.1016/j.jhin.2019.02.019 30826342

[6] The White House. National Action Plan for Combating Antibiotic-Resistant Bacteria. 2015. Available: https://www.cdc.gov/drugresistance/pdf/national_action_plan_for_combating_antibotic-resistant_bacteria.pdf

[7] Davies SC. Annual Report of the Chief Medical Officer, Volume Two, 2011,' ' Infections and the rise of antimicrobial resistance [Internet]. Department of Health; 2013 3 Available: https://www.gov.uk/government/publications/chief-medical-officer-annual-report-volume-2 10.1016/S0140-6736(13)60604-2 23489756

[8] NHS England Contracts and Incentives Team. Quality Premium: 2015/16 guidance for CCGs [Internet]. 3rd ed. 2015 Sep. Available: https://www.england.nhs.uk/resources/resources-for-ccgs/ccg-out-tool/ccg-ois/qual-prem/

[9] English Surveillance Programme forAntimicrobial Utilisation andResistance (ESPAUR) Report 2018. 2018 Oct.

[10] Lienert J, Güdel K, Escher BI. Screening method for ecotoxicological hazard assessment of 42 pharmaceuticals considering human metabolism and excretory routes. Environ Sci Technol. 2007;41: 4471–4478. 10.1021/es0627693 17626454

[11] Besse J-P, Kausch-Barreto C, Garric J. Exposure assessment of pharmaceuticals and their metabolites in the aquatic environment: application to the french situation and preliminary prioritization. Human and Ecological Risk Assessment: An International Journal. 2008;14: 665–695. 10.1080/10807030802235078

[12] Jakobsson HE, Jernberg C, Andersson AF, Sjölund-Karlsson M, Jansson JK, Engstrand L. Short-term antibiotic treatment has differing long-term impacts on the human throat and gut microbiome. PLoS One. 2010;5: e9836 10.1371/journal.pone.0009836 20352091 PMC2844414

[13] Ohlsen K, Ternes T, Werner G, Wallner U, Löffler D, Ziebuhr W, et al Impact of antibiotics on conjugational resistance gene transfer in Staphylococcus aureus in sewage. Environ Microbiol. 2003;5: 711–716. 10.1046/j.1462-2920.2003.00459.x 12871238

[14] Khan S, Beattie TK, Knapp CW. The use of minimum selectable concentrations (MSCs) for determining the selection of antimicrobial resistant bacteria. Ecotoxicology. 2017;26: 283–292. 10.1007/s10646-017-1762-y 28155034 PMC5318476

[15] Baquero F, Coque TM. Widening the spaces of selection: evolution along sublethal antimicrobial gradients. MBio. 2014;5: e02270 10.1128/mBio.02270-14 25491358 PMC4324248

[16] Gullberg E, Albrecht LM, Karlsson C, Sandegren L, Andersson DI. Selection of a multidrug resistance plasmid by sublethal levels of antibiotics and heavy metals. MBio. 2014;5: e01918–14. 10.1128/mBio.01918-14 25293762 PMC4196238

[17] Murray AK, Zhang L, Yin X, Zhang T, Buckling A, Snape J, et al Novel Insights into Selection for Antibiotic Resistance in Complex Microbial Communities. MBio. 2018;9 10.1128/mBio.00969-18 30042197 PMC6058293

[18] Murray AK, Zhang L, Snape J, Gaze WH. Comparing the selective and co-selective effects of different antimicrobials in bacterial communities. Int J Antimicrob Agents. 2019;53: 767–773. 10.1016/j.ijantimicag.2019.03.001 30885807 PMC6546120

[19] Bengtsson-Palme J, Hammarén R, Pal C, Östman M, Björlenius B, Flach C-F, et al Elucidating selection processes for antibiotic resistance in sewage treatment plants using metagenomics. Sci Total Environ. 2016;572: 697–712. 10.1016/j.scitotenv.2016.06.228 27542633

[20] Karkman A, Pärnänen K, Larsson DGJ. Fecal pollution can explain antibiotic resistance gene abundances in anthropogenically impacted environments. Nat Commun. 2019;10: 80 10.1038/s41467-018-07992-3 30622259 PMC6325112

[21] Amos GCA, Gozzard E, Carter CE, Mead A, Bowes MJ, Hawkey PM, et al Validated predictive modelling of the environmental resistome. ISME J. 2015;9: 1467–1476. 10.1038/ismej.2014.237 25679532 PMC4438333

[22] Amos GCA, Ploumakis S, Zhang L, Hawkey PM, Gaze WH, Wellington EMH. The widespread dissemination of integrons throughout bacterial communities in a riverine system. ISME J. 2018;12: 681–691. 10.1038/s41396-017-0030-8 29374269 PMC5864220

[23] Marti E, Jofre J, Balcazar JL. Prevalence of antibiotic resistance genes and bacterial community composition in a river influenced by a wastewater treatment plant. PLoS One. 2013;8: e78906 10.1371/journal.pone.0078906 24205347 PMC3808343

[24] Berglund B, Fick J, Lindgren P-E. Urban wastewater effluent increases antibiotic resistance gene concentrations in a receiving northern European river. Environ Toxicol Chem. 2015;34: 192–196. 10.1002/etc.2784 25331227

[25] Hu A, Ju F, Hou L, Li J, Yang X, Wang H, et al Strong impact of anthropogenic contamination on the co-occurrence patterns of a riverine microbial community. Environ Microbiol. 2017; 10.1111/1462-2920.13942 28967165

[26] Bai Y, Meng W, Xu J, Zhang Y, Guo C. Occurrence, distribution and bioaccumulation of antibiotics in the Liao River Basin in China. Environ Sci Process Impacts. 2014;16: 586–593. 10.1039/c3em00567d 24509869

[27] Sabri NA, Schmitt H, Van der Zaan B, Gerritsen HW, Zuidema T, Rijnaarts HHM, et al Prevalence of antibiotics and antibiotic resistance genes in a wastewater effluent-receiving river in the Netherlands. Journal of Environmental Chemical Engineering. 2018; 10.1016/j.jece.2018.03.004

[28] Gao L, Shi Y, Li W, Liu J, Cai Y. Occurrence, distribution and bioaccumulation of antibiotics in the Haihe River in China. J Environ Monit. 2012;14: 1248–1255. 10.1039/c2em10916f 22402740

[29] Watkinson AJ, Murby EJ, Kolpin DW, Costanzo SD. The occurrence of antibiotics in an urban watershed: from wastewater to drinking water. Sci Total Environ. 2009;407: 2711–2723. 10.1016/j.scitotenv.2008.11.059 19138787

[30] Singer AC, Järhult JD, Grabic R, Khan GA, Lindberg RH, Fedorova G, et al Intra- and inter-pandemic variations of antiviral, antibiotics and decongestants in wastewater treatment plants and receiving rivers. PLoS One. 2014;9: e108621 10.1371/journal.pone.0108621 25254643 PMC4177917

[31] Johnson AC, Keller V, Dumont E, Sumpter JP. Assessing the concentrations and risks of toxicity from the antibiotics ciprofloxacin, sulfamethoxazole, trimethoprim and erythromycin in European rivers. Sci Total Environ. 2015;511: 747–755. 10.1016/j.scitotenv.2014.12.055 25617699

[32] Murray GL, Bradshaw CS, Bissessor M, Danielewski J, Garland SM, Jensen JS, et al Increasing Macrolide and Fluoroquinolone Resistance in Mycoplasma genitalium. Emerging Infect Dis. 2017;23: 809–812. 10.3201/eid2305.161745 28418319 PMC5403035

[33] Sousa JCG, Ribeiro AR, Barbosa MO, Pereira MFR, Silva AMT. A review on environmental monitoring of water organic pollutants identified by EU guidelines. J Hazard Mater. 2017;344: 146–162. 10.1016/j.jhazmat.2017.09.058 29032095

[34] Barbosa MO, Moreira NFF, Ribeiro AR, Pereira MFR, Silva AMT. Occurrence and removal of organic micropollutants: An overview of the watch list of EU Decision 2015/495. Water Res. 2016;94: 257–279. 10.1016/j.watres.2016.02.047 26967909

[35] Keller VDJ, Williams RJ, Lofthouse C, Johnson AC. Worldwide estimation of river concentrations of any chemical originating from sewage-treatment plants using dilution factors. Environ Toxicol Chem. 2014;33: 447–452. 10.1002/etc.2441 24375744 PMC4253128

[36] Singer AC, Nunn MA, Gould EA, Johnson AC. Potential risks associated with the proposed widespread use of Tamiflu. Environ Health Perspect. 2007;115: 102–106. 10.1289/ehp.9574 17366827 PMC1797841

[37] Singer AC, Colizza V, Schmitt H, Andrews J, Balcan D, Huang WE, et al Assessing the ecotoxicologic hazards of a pandemic influenza medical response. Environ Health Perspect. 2011;119: 1084–1090. 10.1289/ehp.1002757 21367688 PMC3237342

[38] Williams RJ, Keller VDJ, Johnson AC, Young AR, Holmes MGR, Wells C, et al A national risk assessment for intersex in fish arising from steroid estrogens. Environ Toxicol Chem. 2009;28: 220–230. 10.1897/08-047.1 18817457

[39] Besse J-P, Garric J. Human pharmaceuticals in surface waters. Implementation of a prioritization methodology and application to the French situation. Toxicol Lett. 2008;176: 104–123. 10.1016/j.toxlet.2007.10.012 18077113

[40] Carvalho RN, Ceriani L, Ippolito A, Lettieri T. JRC Technical Report: Development of the first Watch List under the Environmental Quality Standards Directive (EUR27142). European Commission. 2015;

[41] Brogard JM, Jehl F, Blickle JF, Arnaud JP, Monteil H. [The role of ciprofloxacine metabolites in its biliary and urinary elimination in man]. Pathol Biol. 1988;36: 719–723. 3054754

[42] Zhang Q-Q, Ying G-G, Pan C-G, Liu Y-S, Zhao J-L. Comprehensive evaluation of antibiotics emission and fate in the river basins of China: source analysis, multimedia modeling, and linkage to bacterial resistance. Environ Sci Technol. 2015;49: 6772–6782. 10.1021/acs.est.5b00729 25961663

[43] Kümmerer K, Henninger A. Promoting resistance by the emission of antibiotics from hospitals and households into effluent. Clin Microbiol Infect. 2003;9: 1203–1214. 10.1111/j.1469-0691.2003.00739.x 14686985

[44] Monteiro SC, Boxall ABA. Occurrence and fate of human pharmaceuticals in the environment. Rev Environ Contam Toxicol. 2010;202: 53–154. 10.1007/978-1-4419-1157-5_2 19898761

[45] Nightingale CH. Moxifloxacin, a new antibiotic designed to treat community-acquired respiratory tract infections: a review of microbiologic and pharmacokinetic-pharmacodynamic characteristics. Pharmacotherapy. 2000;20: 245–256. 10.1592/phco.20.4.245.34880 10730681

[46] Lindberg RH, Wennberg P, Johansson MI, Tysklind M, Andersson BAV. Screening of human antibiotic substances and determination of weekly mass flows in five sewage treatment plants in Sweden. Environ Sci Technol. 2005;39: 3421–3429. 10.1021/es048143z 15952345

[47] Jia A, Wan Y, Xiao Y, Hu J. Occurrence and fate of quinolone and fluoroquinolone antibiotics in a municipal sewage treatment plant. Water Res. 2012;46: 387–394. 10.1016/j.watres.2011.10.055 22118907

[48] Göbel A, McArdell CS, Joss A, Siegrist H, Giger W. Fate of sulfonamides, macrolides, and trimethoprim in different wastewater treatment technologies. Sci Total Environ. 2007;372: 361–371. 10.1016/j.scitotenv.2006.07.039 17126383

[49] Radjenović J, Petrović M, Barceló D. Fate and distribution of pharmaceuticals in wastewater and sewage sludge of the conventional activated sludge (CAS) and advanced membrane bioreactor (MBR) treatment. Water Res. 2009;43: 831–841. 10.1016/j.watres.2008.11.043 19091371

[50] Watkinson AJ, Murby EJ, Costanzo SD. Removal of antibiotics in conventional and advanced wastewater treatment: implications for environmental discharge and wastewater recycling. Water Res. 2007;41: 4164–4176. 10.1016/j.watres.2007.04.005 17524445

[51] Michael I, Rizzo L, McArdell CS, Manaia CM, Merlin C, Schwartz T, et al Urban wastewater treatment plants as hotspots for the release of antibiotics in the environment: a review. Water Res. 2013;47: 957–995. 10.1016/j.watres.2012.11.027 23266388

[52] Johnson AC, Jürgens MD, Nakada N, Hanamoto S, Singer AC, Tanaka H. Linking changes in antibiotic effluent concentrations to flow, removal and consumption in four different UK sewage treatment plants over four years. Environ Pollut. 2017;220: 919–926. 10.1016/j.envpol.2016.10.077 27839989

[53] Keller V, Young AR. Development of an Integrated Water Resources and Water Quality Modelling System. 2004;

[54] Young AR, Grew R, Holmes MG. Low Flows 2000: a national water resources assessment and decision support tool. Water Sci Technol. 2003;48: 119–126. 15137161

[55] Bengtsson-Palme J, Larsson DGJ. Concentrations of antibiotics predicted to select for resistant bacteria: Proposed limits for environmental regulation. Environ Int. 2016;86: 140–149. 10.1016/j.envint.2015.10.015 26590482

[56] Sandegren L. Selection of antibiotic resistance at very low antibiotic concentrations. Ups J Med Sci. 2014;119: 103–107. 10.3109/03009734.2014.904457 24694026 PMC4034545

[57] Strukova EN, Portnoy YA, Zinner SH, Firsov AA. Predictors of bacterial resistance using in vitro dynamic models: area under the concentration-time curve related to either the minimum inhibitory or mutant prevention antibiotic concentration. J Antimicrob Chemother. 2016;71: 678–684. 10.1093/jac/dkv387 26626718

[58] Gullberg E, Cao S, Berg OG, Ilbäck C, Sandegren L, Hughes D, et al Selection of resistant bacteria at very low antibiotic concentrations. PLoS Pathog. 2011;7: e1002158 10.1371/journal.ppat.1002158 21811410 PMC3141051

[59] Liu A, Fong A, Becket E, Yuan J, Tamae C, Medrano L, et al Selective advantage of resistant strains at trace levels of antibiotics: a simple and ultrasensitive color test for detection of antibiotics and genotoxic agents. Antimicrob Agents Chemother. 2011;55: 1204–1210. 10.1128/AAC.01182-10 21199928 PMC3067110

[60] Andersson DI, Hughes D. Microbiological effects of sublethal levels of antibiotics. Nat Rev Microbiol. 2014;12: 465–478. 10.1038/nrmicro3270 24861036

[61] Andersson DI, Hughes D. Evolution of antibiotic resistance at non-lethal drug concentrations. Drug Resist Updat. 2012;15: 162–172. 10.1016/j.drup.2012.03.005 22516308

[62] Mezger A, Gullberg E, Göransson J, Zorzet A, Herthnek D, Tano E, et al A general method for rapid determination of antibiotic susceptibility and species in bacterial infections. J Clin Microbiol. 2015;53: 425–432. 10.1128/JCM.02434-14 25411178 PMC4298551

[63] Hughes D, Andersson DI. Selection of resistance at lethal and non-lethal antibiotic concentrations. Curr Opin Microbiol. 2012;15: 555–560. 10.1016/j.mib.2012.07.005 22878455

[64] Kraupner N, Ebmeyer S, Bengtsson-Palme J, Fick J, Kristiansson E, Flach C-F, et al Selective concentration for ciprofloxacin resistance in' ' Escherichia coli grown in complex aquatic bacterial biofilms. Environ Int. 2018;116: 255–268. 10.1016/j.envint.2018.04.029 29704804

[65] AMR Industry Alliance. AMR Industry Alliance Antibiotic Discharge Targets. In: AMR Alliance Recommended PNECs for Risk Assessments [Internet]. 2018 [cited 14 Jan 2019]. Available: https://www.amrindustryalliance.org/wp-content/uploads/2018/09/AMR_Industry_Alliance_List-of-Predicted-No-Effect-Concentrations-PNECs.pdf

[66] Singer A, Shaw H, Rhodes V, Hart A. Review of antimicrobial resistance in the environment and its relevance to environmental regulators. Front Microbiol. 2016;7: 1728 10.3389/fmicb.2016.01728 27847505 PMC5088501

[67] Bürgmann H, Frigon D, H Gaze W, M Manaia C, Pruden A, Singer AC, et al Water and sanitation: an essential battlefront in the war on antimicrobial resistance. FEMS Microbiol Ecol. 2018;94 10.1093/femsec/fiy101 29878227

[68] International Environmental AMR Forum. Initiatives for Addressing Antimicrobial Resistance in the Environment Current: Situation and Challenges. Wellcome Trust, U.S. CDC, UK Science & Innovation Network; 2018. Available: https://wellcome.ac.uk/sites/default/files/antimicrobial-resistance-environment-report.pdf

[69] Gouliouris T, Raven KE, Moradigaravand D, Ludden C, Coll F, Blane B, et al Detection of vancomycin-resistant Enterococcus faecium hospital-adapted lineages in municipal wastewater treatment plants indicates widespread distribution and release into the environment. Genome Res. 2019;29: 626–634. 10.1101/gr.232629.117 30898881 PMC6442392

[70] Runcharoen C, Moradigaravand D, Blane B, Paksanont S, Thammachote J, Anun S, et al Whole genome sequencing reveals high-resolution epidemiological links between clinical and environmental Klebsiella pneumoniae. Genome Med. 2017;9: 6 10.1186/s13073-017-0397-1 28118859 PMC5264300

[71] Pärnänen KMM, Narciso-da-Rocha C, Kneis D, Berendonk TU, Cacace D, Do TT, et al Antibiotic resistance in European wastewater treatment plants mirrors the pattern of clinical antibiotic resistance prevalence. Sci Adv. 2019;5: eaau9124 10.1126/sciadv.aau9124 30944853 PMC6436925

[72] Pruden A, Pei R, Storteboom H, Carlson KH. Antibiotic resistance genes as emerging contaminants: studies in northern Colorado. Environ Sci Technol. 2006;40: 7445–7450. 10.1021/es060413l 17181002

[73] Gillings MR. DNA as a pollutant: the clinical class 1 integron. Curr Pollution Rep. 2018;4: 49–55. 10.1007/s40726-018-0076-x

[74] Jang J, Hur HG, Sadowsky MJ, Byappanahalli MN, Yan T, Ishii S. Environmental Escherichia coli: ecology and public health implications-a review. J Appl Microbiol. 2017;123: 570–581. 10.1111/jam.13468 28383815

[75] McCann CM, Christgen B, Roberts JA, Su J-Q, Arnold KE, Gray ND, et al Understanding drivers of antibiotic resistance genes in High Arctic soil ecosystems. Environ Int. 2019;125: 497–504. 10.1016/j.envint.2019.01.034 30700387

[76] Furness LE, Campbell A, Zhang L, Gaze WH, McDonald RA. Wild small mammals as sentinels for the environmental transmission of antimicrobial resistance. Environ Res. 2017;154: 28–34. 10.1016/j.envres.2016.12.014 28013185

[77] Amos GCA, Hawkey PM, Gaze WH, Wellington EM. Waste water effluent contributes to the dissemination of CTX-M-15 in the natural environment. J Antimicrob Chemother. 2014;69: 1785–1791. 10.1093/jac/dku079 24797064 PMC4054988

[78] Chait R, Palmer AC, Yelin I, Kishony R. Pervasive selection for and against antibiotic resistance in inhomogeneous multistress environments. Nat Commun. 2016;7: 10333 10.1038/ncomms10333 26787239 PMC4735756

[79] Zhang Y, Gu AZ, Cen T, Li X, He M, Li D, et al Sub-inhibitory concentrations of heavy metals facilitate the horizontal transfer of plasmid-mediated antibiotic resistance genes in water environment. Environ Pollut. 2018;237: 74–82. 10.1016/j.envpol.2018.01.032 29477117

[80] Dong P, Wang H, Fang T, Wang Y, Ye Q. Assessment of extracellular antibiotic resistance genes (eARGs) in typical environmental samples and the transforming ability of eARG. Environ Int. 2019;125: 90–96. 10.1016/j.envint.2019.01.050 30711653

[81] O’Brien TF. Emergence, spread, and environmental effect of antimicrobial resistance: how use of an antimicrobial anywhere can increase resistance to any antimicrobial anywhere else. Clin Infect Dis. 2002;34 Suppl 3: S78–84. 10.1086/340244 11988877

[82] Li R, Yu H, Xie M, Chen K, Dong N, Lin D, et al Genetic basis of chromosomally-encoded mcr-1 gene. Int J Antimicrob Agents. 2018;51: 578–585. 10.1016/j.ijantimicag.2017.11.015 29197647

[83] Cottell JL, Webber MA, Coldham NG, Taylor DL, Cerdeño-Tárraga AM, Hauser H, et al Complete sequence and molecular epidemiology of IncK epidemic plasmid encoding blaCTX-M-14. Emerging Infect Dis. 2011;17: 645–652. 10.3201/eid1704.101009 21470454 PMC3377399

[84] Valverde A, Cantón R, Garcillán-Barcia MP, Novais A, Galán JC, Alvarado A, et al Spread of bla(CTX-M-14) is driven mainly by IncK plasmids disseminated among Escherichia coli phylogroups A, B1, and D in Spain. Antimicrob Agents Chemother. 2009;53: 5204–5212. 10.1128/AAC.01706-08 19786598 PMC2786348

[85] Bai F, Li X, Niu B, Zhang Z, Malakar PK, Liu H, et al A mcr-1-Carrying Conjugative IncX4 Plasmid in Colistin-Resistant Escherichia coli ST278 Strain Isolated From Dairy Cow Feces in Shanghai, China. Front Microbiol. 2018;9: 2833 10.3389/fmicb.2018.02833 30559724 PMC6287198

[86] Hernando-Amado S, Sanz-García F, Blanco P, Martínez JL. Fitness costs associated with the acquisition of antibiotic resistance. Essays Biochem. 2017;61: 37–48. 10.1042/EBC20160057 28258228

[87] Durão P, Balbontín R, Gordo I. Evolutionary mechanisms shaping the maintenance of antibiotic resistance. Trends Microbiol. 2018;26: 677–691. 10.1016/j.tim.2018.01.005 29439838

[88] Dhanji H, Murphy NM, Akhigbe C, Doumith M, Hope R, Livermore DM, et al Isolation of fluoroquinolone-resistant O25b:H4-ST131 Escherichia coli with CTX-M-14 extended-spectrum β-lactamase from UK river water. J Antimicrob Chemother. 2011;66: 512–516. 10.1093/jac/dkq472 21172781

[89] Lehmann K, Bell T, Bowes MJ, Amos GCA, Gaze WH, Wellington EMH, et al Trace levels of sewage effluent are sufficient to increase class 1 integron prevalence in freshwater biofilms without changing the core community. Water Res. 2016;106: 163–170. 10.1016/j.watres.2016.09.035 27710799

[90] Song Y, Cui L, López JÁS, Xu J, Zhu Y-G, Thompson IP, et al Raman-Deuterium Isotope Probing for in-situ identification of antimicrobial resistant bacteria in Thames River. Sci Rep. 2017;7: 16648 10.1038/s41598-017-16898-x 29192181 PMC5709456

[91] Dhanji H, Doumith M, Rooney PJ, O’Leary MC, Loughrey AC, Hope R, et al Molecular epidemiology of fluoroquinolone-resistant ST131 Escherichia coli producing CTX-M extended-spectrum beta-lactamases in nursing homes in Belfast, UK. J Antimicrob Chemother. 2011;66: 297–303. 10.1093/jac/dkq463 21131323

[92] Weingarten RA, Johnson RC, Conlan S, Ramsburg AM, Dekker JP, Lau AF, et al Genomic analysis of hospital plumbing reveals diverse reservoir of bacterial plasmids conferring carbapenem resistance. MBio. 2018;9 10.1128/mBio.02011-17 29437920 PMC5801463

[93] Paulus GK, Hornstra LM, Alygizakis N, Slobodnik J, Thomaidis N, Medema G. The impact of on-site hospital wastewater treatment on the downstream communal wastewater system in terms of antibiotics and antibiotic resistance genes. Int J Hyg Environ Health. 2019;1 10.1016/j.ijheh.2019.01.004 30737165

[94] McNulty CAM, Lecky DM, Xu-McCrae L, Nakiboneka-Ssenabulya D, Chung K-T, Nichols T, et al CTX-M ESBL-producing Enterobacteriaceae: estimated prevalence in adults in England in 2014. J Antimicrob Chemother. 2018;73: 1368–1388. 10.1093/jac/dky007 29514211 PMC5909627

[95] Dhanji H, Patel R, Wall R, Doumith M, Patel B, Hope R, et al Variation in the genetic environments of bla(CTX-M-15) in Escherichia coli from the faeces of travellers returning to the United Kingdom. J Antimicrob Chemother. 2011;66: 1005–1012. 10.1093/jac/dkr041 21393166

[96] Brochado AR, Telzerow A, Bobonis J, Banzhaf M, Mateus A, Selkrig J, et al Species-specific activity of antibacterial drug combinations. Nature. 2018;559: 259–263. 10.1038/s41586-018-0278-9 29973719 PMC6219701

[97] Tekin E, White C, Kang TM, Singh N, Cruz-Loya M, Damoiseaux R, et al Prevalence and patterns of higher-order drug interactions in Escherichia coli. npj Syst Biol Appl. 2018;4: 31 10.1038/s41540-018-0069-9 30181902 PMC6119685

[98] European Centre for Disease Prevention and Control (ECDC), European Food Safety Authority (EFSA), European Medicines Agency (EMA). ECDC/EFSA/EMA second joint report on the integrated analysis of the consumption of antimicrobial agents and occurrence of antimicrobial resistance in bacteria from humans and food‐producing animals. EFSA Journal. 2017;15 10.2903/j.efsa.2017.4872 PMC7009874 32625542

[99] European Commission. SCENIHR (Scientific Committee on Emerging and Newly Identified Health Risks), Research strategy to address the knowledge gaps on the antimicrobial resistance effects of biocides,. 2010;

[100] Carey DE, McNamara PJ. The impact of triclosan on the spread of antibiotic resistance in the environment. Front Microbiol. 2014;5: 780 10.3389/fmicb.2014.00780 25642217 PMC4295542

[101] Veterinary Medicines Directorate. UK Veterinary Antibiotic' ' Resistance and Sales Surveillance Report UK—VARSS 2017. Veterinary Medicines Directorate; 2018 Oct.

[102] Baker-Austin C, Wright MS, Stepanauskas R, McArthur JV. Co-selection of antibiotic and metal resistance. Trends Microbiol. 2006;14: 176–182. 10.1016/j.tim.2006.02.006 16537105

[103] Seiler C, Berendonk TU. Heavy metal driven co-selection of antibiotic resistance in soil and water bodies impacted by agriculture and aquaculture. Front Microbiol. 2012;3: 399 10.3389/fmicb.2012.00399 23248620 PMC3522115

[104] Beane SJ, Comber SDW, Rieuwerts J, Long P. Abandoned metal mines and their impact on receiving waters: A case study from Southwest England. Chemosphere. 2016;153: 294–306. 10.1016/j.chemosphere.2016.03.022 27023117

[105] Comber SDW, Gardner MJ, Churchley J. Aluminium speciation: implications of wastewater effluent dosing on river water quality. Chem Spec & Bioavail. 2005;17: 117–128. 10.3184/095422905782774874

[106] Wang Y, Lu J, Mao L, Li J, Yuan Z, Bond PL, et al Antiepileptic drug carbamazepine promotes horizontal transfer of plasmid-borne multi-antibiotic resistance genes within and across bacterial genera. ISME J. 2018;13: 509–522. 10.1038/s41396-018-0275-x 30291330 PMC6331567

[107] Comber S, Gardner M, Sörme P, Leverett D, Ellor B. Active pharmaceutical ingredients entering the aquatic environment from wastewater treatment works: A cause for concern? Sci Total Environ. 2017;613–614: 538–547. 10.1016/j.scitotenv.2017.09.101 28926808

[108] Kurenbach B, Marjoshi D, Amábile-Cuevas CF, Ferguson GC, Godsoe W, Gibson P, et al Sublethal exposure to commercial formulations of the herbicides dicamba, 2,4-dichlorophenoxyacetic acid, and glyphosate cause changes in antibiotic susceptibility in Escherichia coli and Salmonella enterica serovar Typhimurium. MBio. 2015;6 10.1128/mBio.00009-15 25805724 PMC4453521

[109] Kurenbach B, Gibson PS, Hill AM, Bitzer AS, Silby MW, Godsoe W, et al Herbicide ingredients change Salmonella enterica sv. Typhimurium and Escherichia coli antibiotic responses. Microbiology (Reading, Engl). 2017; 10.1099/mic.0.000573 29139345 PMC5845734

[110] Johnson AC, Acreman MC, Dunbar MJ, Feist SW, Giacomello AM, Gozlan RE, et al The British river of the future: how climate change and human activity might affect two contrasting river ecosystems in England. Sci Total Environ. 2009;407: 4787–4798. 10.1016/j.scitotenv.2009.05.018 19505713

[111] Keller VDJ, Lloyd P, Terry JA, Williams RJ. Impact of climate change and population growth on a risk assessment for endocrine disruption in fish due to steroid estrogens in England and Wales. Environ Pollut. 2015;197: 262–268. 10.1016/j.envpol.2014.11.017 25440503

[112] Johnson AC, Williams RJ. A Model To Estimate Influent and Effluent Concentrations of Estradiol, Estrone, and Ethinylestradiol at Sewage Treatment Works. Environ Sci Technol. 2004;38: 3649–3658. 10.1021/es035342u 15296317

[113] Kugathas S, Williams RJ, Sumpter JP. Prediction of environmental concentrations of glucocorticoids: the River Thames, UK, as an example. Environ Int. 2012;40: 15–23. 10.1016/j.envint.2011.11.007 22280923

[114] Johnson AC, Jürgens MD, Williams RJ, Kümmerer K, Kortenkamp A, Sumpter JP. Do cytotoxic chemotherapy drugs discharged into rivers pose a risk to the environment and human health? An overview and UK case study. J Hydrol (Amst). 2008;348: 167–175. 10.1016/j.jhydrol.2007.09.054

[115] Davies SC. Reducing inappropriate prescribing of antibiotics in English primary care: evidence and outlook. J Antimicrob Chemother. 2018;73: 833–834. 10.1093/jac/dkx535 29490040

[116] Finistrella V, Purslow C. Advisory Committee on Antimicrobial Prescribing, Resistance and Healthcare Associated Infection (APRHAI). 8th Annual Report, April 2016—March 2017. Department of Health and Social Care; 2017.

[117] Smieszek T, Pouwels KB, Dolk FCK, Smith DRM, Hopkins S, Sharland M, et al Potential for reducing inappropriate antibiotic prescribing in English primary care. J Antimicrob Chemother. 2018;73: ii36–ii43. 10.1093/jac/dkx500 29490058 PMC5890667

[118] Pouwels KB, Dolk FCK, Smith DRM, Robotham JV, Smieszek T. Actual versus “ideal” antibiotic prescribing for common conditions in English primary care. J Antimicrob Chemother. 2018;73: 19–26. 10.1093/jac/dkx502 29490060 PMC5890776

[119] Harris CA, Hamilton PB, Runnalls TJ, Vinciotti V, Henshaw A, Hodgson D, et al The consequences of feminization in breeding groups of wild fish. Environ Health Perspect. 2011;119: 306–311. 10.1289/ehp.1002555 21362587 PMC3059991

[120] Johnson AC, Dumont E, Williams RJ, Oldenkamp R, Cisowska I, Sumpter JP. Do concentrations of ethinylestradiol, estradiol, and diclofenac in European rivers exceed proposed EU environmental quality standards? Environ Sci Technol. 2013;47: 12297–12304. 10.1021/es4030035 24074201

[121] Jobling S, Williams R, Johnson A, Taylor A, Gross-Sorokin M, Nolan M, et al Predicted exposures to steroid estrogens in U.K. rivers correlate with widespread sexual disruption in wild fish populations. Environ Health Perspect. 2006;114 Suppl 1: 32–39. 10.1289/ehp.8050 16818244 PMC1874167

[122] Owen R, Jobling S. Environmental science: The hidden costs of flexible fertility. Nature. 2012;485: 441 10.1038/485441a 22622553

[123] Yazdankhah SP, Scheie AA, Høiby EA, Lunestad B-T, Heir E, Fotland TØ, et al Triclosan and antimicrobial resistance in bacteria: an overview. Microb Drug Resist. 2006;12: 83–90. 10.1089/mdr.2006.12.83 16922622

[124] Kampf G. Antibiotic ResistanceCan Be Enhanced in Gram-Positive Species by Some Biocidal Agents Used for Disinfection. Antibiotics (Basel). 2019;8 10.3390/antibiotics8010013 30717270 PMC6466599

[125] Di Cesare A, Eckert E, Corno G. Co-selection of antibiotic and heavy metal resistance in freshwater bacteria. J Limnol. 2016;75 10.4081/jlimnol.2016.1198

